# Supplementary figures and images for: Attentional shift within and between faces: Evidence from children with and without a diagnosis of autism spectrum disorder
Source: PLoS One. 2021 May 14;16(5):e0251475. doi: 10.1371/journal.pone.0251475 (PMC8121363; doi:10.1371/journal.pone.0251475)

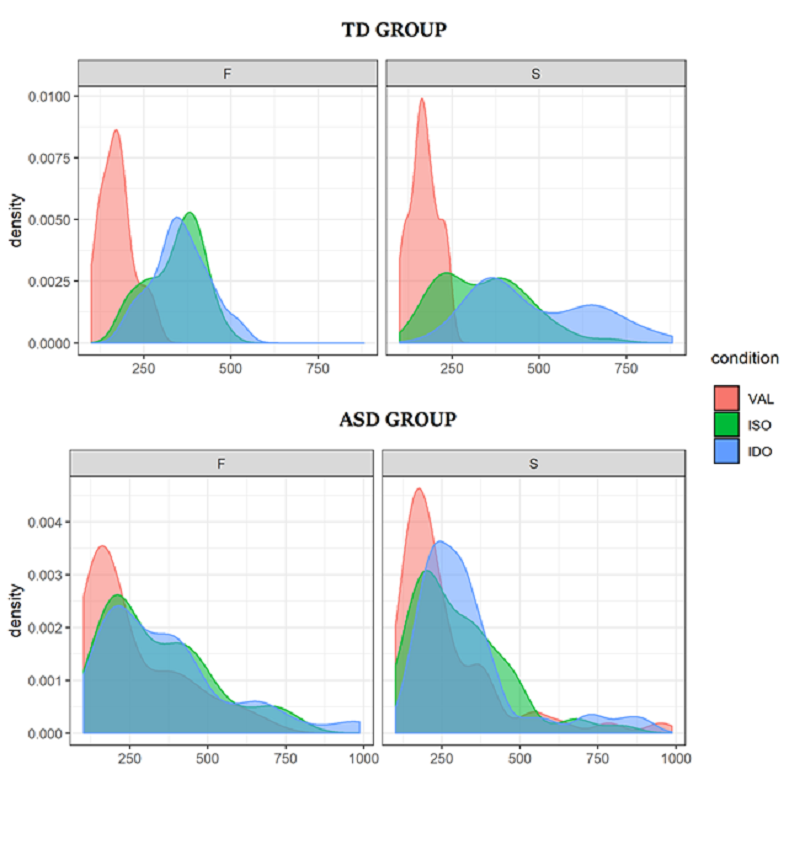

Supplement: S1 Fig — Target detection times in milliseconds per group i.e. TD and ASD, stimulus i.e. phase scrambled stimuli (S) or Face (F) and conditions, i.e. VAL, ISO and IDO. (TIF) [file pone.0251475.s001.tif]

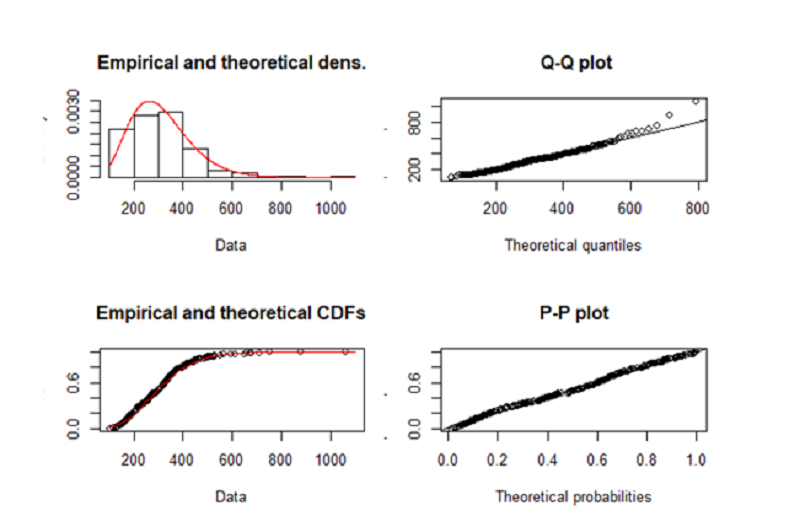

Supplement: S2 Fig — Residual distribution for detection times (in milliseconds) to non-censored data i.e. Gamma, by maximum likelihood (mle). (TIF) [file pone.0251475.s002.tif]

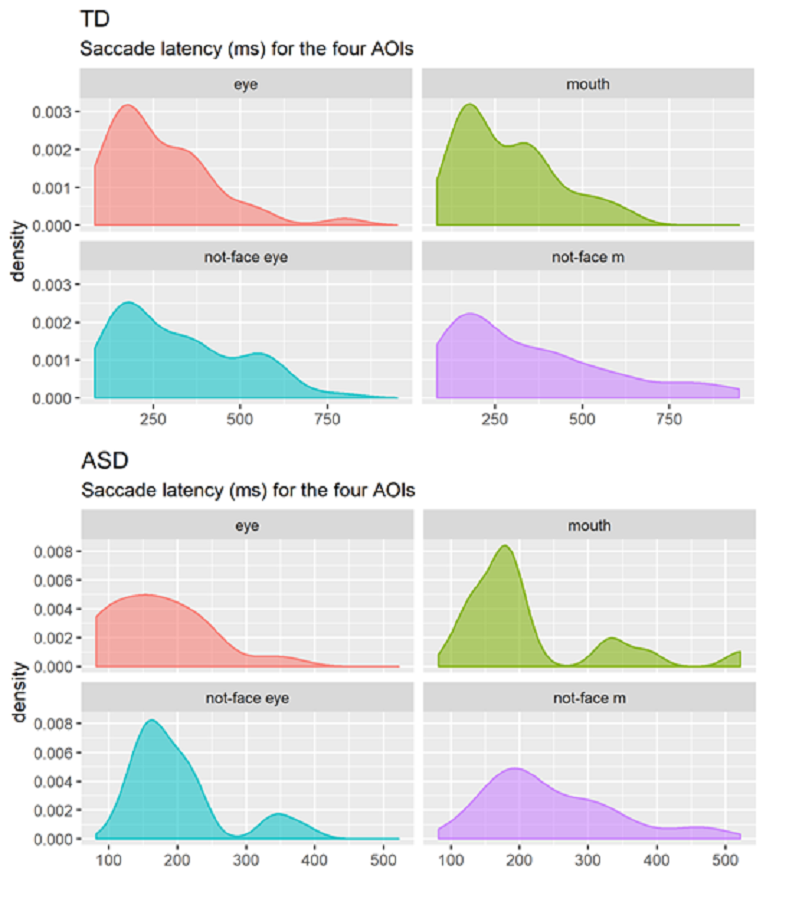

Supplement: S3 Fig — Target detection times in milliseconds per group i.e., TD and ASD. Moreover, considering four AOIs i.e., eye = upper face, mouth = lower face, not-face eye = upper scrambled-phase, not-face m = lower scrambled-phase stimulus. Plots reflect only a sub-sample of participants (12 TD and 7 ASD) that reached at least three valid trials for each AOIs (in each condition and for each stimulus). (TIF) [file pone.0251475.s003.tif]
