# Supplementary material for: Attentional shift within and between faces: Evidence from children with and without a diagnosis of autism spectrum disorder
Source: PLoS One. 2021 May 14;16(5):e0251475. doi: 10.1371/journal.pone.0251475 (PMC8121363; doi:10.1371/journal.pone.0251475)
Supplement: Attachment — Submitted filename: Response to Reviewers.pdf [file pone.0251475.s005.pdf]

Reviewer #1:

The study used remote eye-tracking and employed Egly’s paradigm to examine space-based and object-based attention in children with and without ASD. Egly’s paradigm was adapted to show either two identical faces (side by side) or their phase-scrambled versions. The authors reported group differences in object-based – but not space-based – attention: the TD group was faster to detect a target when it was presented in the cued location (i.e., cue and target appeared in the same phase-scrambled stimulus) compared to the un-cued location (i.e., cue and target did not appear in the same phase-scrambled stimulus). The ASD group did not show this pattern. This finding was only evident in the phase-scrambled but not the face condition.

The topic of the paper is multifaceted, the authors employed an interesting experimental paradigm to examine space- vs object-based attention, and the study findings would be of interest to a wide readership. However, some clarifications are required, with my main comments relating to the study motivations, data quality, and wider theoretical implications of the findings.

Major comments

I viewed the csv data sheet in the OSF repository, and the first names of participants were included in the file alongside their age, sex, and ASD diagnosis. The name column urgently needs to be removed. (The readme.txt file also does not seem to correspond with the csv file.)

Response: **We apologize for the inconvenience. We removed the name column from the csv file. We also modified the readme.txt file that now presents the essential information to read the target_detection_time_TD_ASD.csv file.**

The authors present relevant literature in their introduction, though the rationale for the present study was still a bit unclear, so that it was not entirely clear why Egly’s paradigm in particular was suitable. For example, while the introduction mentions studies on visual orienting behaviour toward social stimuli in ASD, the current paradigm does not contain competing visual information since only two faces are shown (or their phase-scrambled equivalent). The paper states as an aim “deepen[ing] the understanding of how attentional mechanisms are difunctional in ASD compared with TD in order to offer new useful tools able to hack specific pattern of attention deployment in children with a diagnosis of ASD”, but this should be more specific. It could be helpful to expand on the literature on attentional difficulties in ASD and/or be more explicit about the study aims by elaborating on why space- vs object-based attention specifically requires investigation to better understand social attention in ASD, which would then motivate Egly’s paradigm as well.

Response: **By following Reviewer 1's suggestion, we extensively revised the introduction by sharply focusing on the reasons why Egly's paradigm might be a relevant tool to understand social attention in ASD, pp. 1-5 (revision are marked in yellow). Indeed this paradigm is suitable for evaluating costs and benefits in dislocating attention toward different attentional focuses. When the attentional focus of visual orienting is a particular region in the visual space (i.e., space-based attention), whatever falls within the focused spatial region, be it an object, a group of objects, or nothing at all, is processed more efficiently than stimuli in non-selected regions (e.g., Cave & Bichot, 1999; Posner, 1980). Conversely, when the attentional focus of visual orienting is an object (i.e. object-based attention), the target of attention is not an arbitrary region, but it is exactly those region that corresponds to candidate objects (Driver & Baylis, 1998; Duncan, 1984). For object-based attention to be deployed, a robust object representation must be established (Chen , 2012). Thus, variables that affect the quality of object representations also influence the degree to which object-based attention is employed. Unlike space-based attention, only object-based attention is a gateway to investigate the complex interplay underling the impact of the selected information (object representation) on the deployment of attentional strategies (Driver, Davis, Russell, Turatto, & Freeman, 2001). So this paradigm evaluates costs and benefits in dislocating attention toward different attentional focuses and we believe that it suits for individuate which conditions and stimuli trigger different attentional strategies in children with a diagnosis of ASD compared to children without a diagnosis of ASD.**

Did data quality differ between groups and were there any calibration requirements or data quality metrics used? This could be particularly relevant also for the ASD group, especially if their data quality was lower and led to more excluded trials. For example, Fig3 illustrates much less consistent data patterns for the ASD than TD group, but is this the result of greater heterogeneity in the ASD group or could this also be down to systematic data quality issues?

Response: **To better clarify what we mean by *data quality*, we enclose two images reporting our minimum calibration requirements. Figure A (below reported) shows two visual examples of valid calibration outputs that met the requirements, resulting in a strict inclusion criterion for all participants. To be considered valid the output should show that the five calibration points were correctly reached at least for one eye, and also the tracked fixations should fall within the calibration's AOI (grey circles) as signaled by the green lines whereas red lines signaled an invalid calibration output.**

Figure A. **Two examples of valid (left side) and invalid (right side) calibration output. Criteria were the same for both groups.**

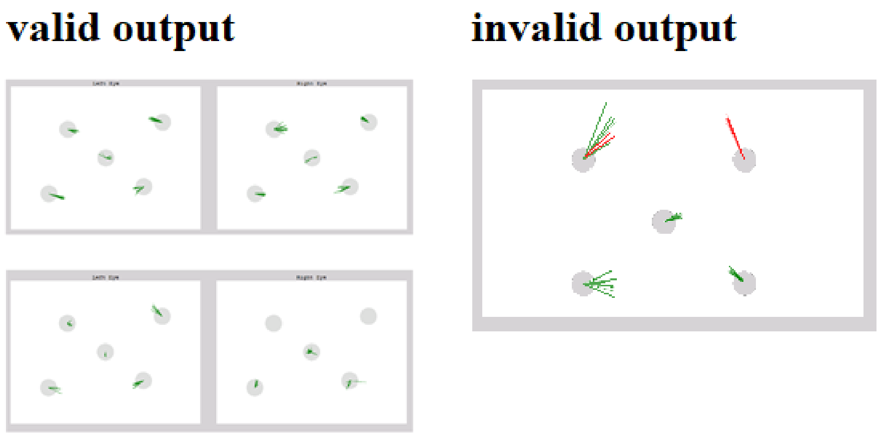

Moreover, the figure B (below reported) shows the mean number of trials by participants split for group (ASD and TD), condition (valid, invalid same object and invalid different object), and stimulus (face and their phase-scrambled version). The two groups did not show a substantial difference in terms of the number of trials per each level of our design. Likely, the huge variability found in the ASD compared with the TD group does not depend on data quality issues but rather on the actual variability shown in target detection by children with a diagnosis of ASD (see also response to the point below).

Figure B. **Mean number of trials and associated 95% CI by participants across groups, condition and stimuli.**

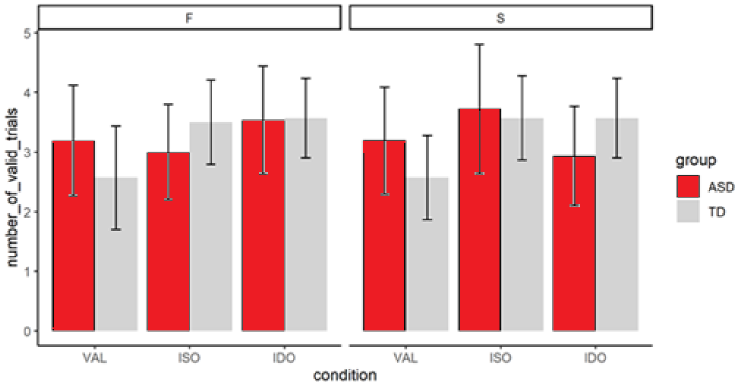

On a note, we will insert all the figures presented in the following responses in the main text if the Reviewer or the Editor suggests doing so.

Related to the point above, there were some critical observations in the data that were not discussed. For instance, the heterogeneity in the ASD group is quite remarkable compared to the TD group (Fig3), but it seems that this was not highlighted anywhere. The ASD group also seems overall slower compared to the TD group, including in the valid condition, which has also not been mentioned (and could also be related to data quality).

Response: **We thank the Reviewer 1 for catching this important point. We think that such heterogeneity do not depends on data quality issues (see response above). Indeed, the heterogeneity is likely to speak for huge variability usually found in individuals with a diagnosis of ASD (Happé, Ronald, Plomin, 2006; Del Bianco et al., 2020). We enclosed in the revised version of the ms these considerations by referring to the heterogeneity found in the ASD group in the Discussion section p. 14.**

Moreover, as requested, we mentioned the difference between the two groups in the valid condition, p. 10. Notably, such a difference (TD were faster than ASD in the valid condition) did not impact the space-based effect that we found in the ASD as well as in the TD group.

Some critical details on the study methodology would need to be included to ensure data integrity.
- It is stated that groups were matched on chronological age, but the ASD group is on average more than 1 year younger – is this an error, how were they age-matched?

Response: **To answer to this issue we clearly show the distribution of participants by group and age in months. Figure C.1 (below) shows that the two groups were not perfectly matched due to the exclusion of participants for poor calibration data, as described in the participants section.**

Figure C. **1) the total number of participants per age (in months) and group.**

1.

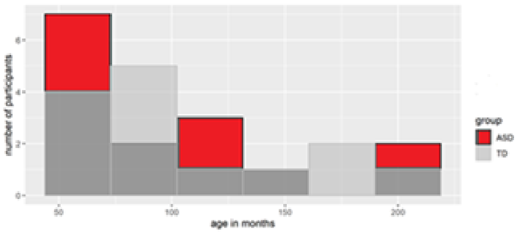

Nevertheless, age (in months) and sex matches between groups, seems to be sufficiently adequate as indicated by Figure C.2.

Figure C. **2) frequency plot for age (in months), sex and group. The dotted lines indicate medians of age by sex (female green and male blue) and group (red ASD and gray TD). No substantial difference emerged by comparing age among female and male participants between groups.**

2.

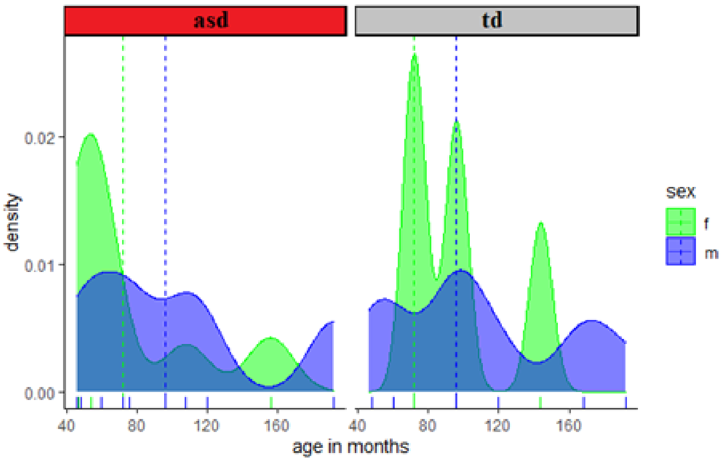

This conclusion is also supported by Figure D (below) showing the scatter plots with the Spearman's correlation coefficient and p-values between age (continuous variable in months) and target detection time (in milliseconds) for both groups (AD and TD) and stimuli (Face and Scrambled version). No association between age and target detection times emerged. Dots are datapoints per single trial.

Figure D **shows the scatter plots with the Spearman's correlation coefficient and p-values between age (continuous variable in months) and target detection time (in milliseconds) for both groups (AD and TD) and stimuli (Face and Scrambled version). Dots indicate target detection times at the trial level.**

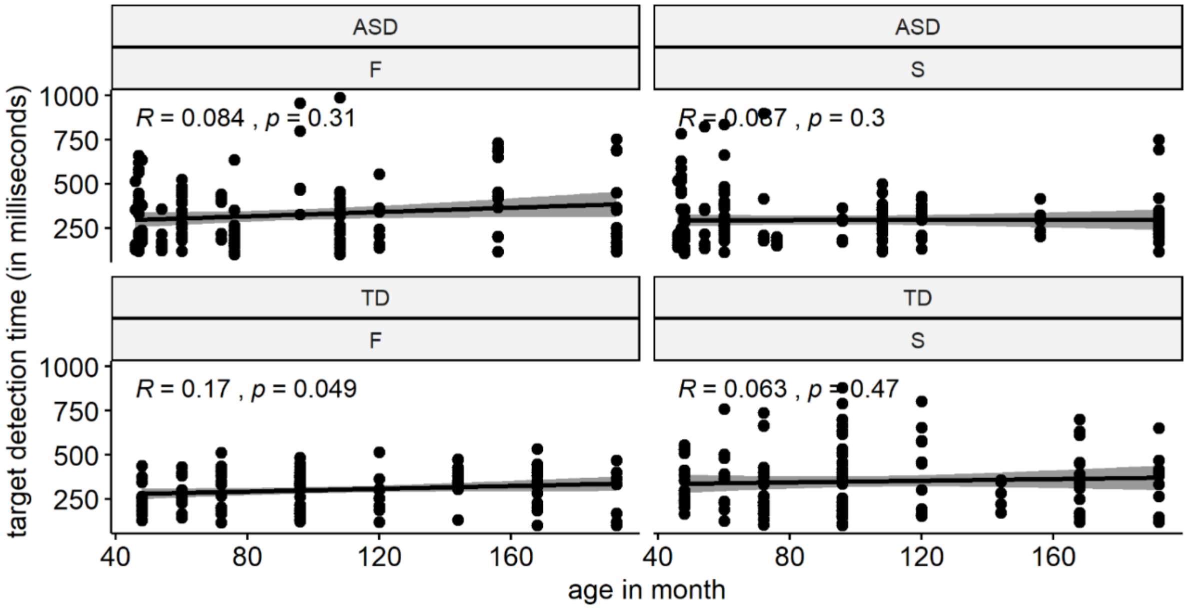

Accordingly, we dedicated a short paragraph in the result section to explain that our results are not better explained by age pp. 11: "[…] Finally, age (treated as a continuos variable) did not show to predict any substantial effect. This result suggests that the effects found in our study likely detected a difference at the group level because our analysis is suitable to estimate effect at both group and participant levels, in an object-based attention task (Pilz, et al., 2012)."

- Why were only 14 TD participants included, what happened to the other 10 who were tested?

Response: **Data on TD participant was collected in class-rooms at schools. We evaluated all children whose parents signed informed consent but TD children who did not matched the ASD group in terms of age and sex were not included in the analysis. We specified this criterion in the revised participants section pp. 6.**

- What was the motivation to include such a wide age range and collapsing all participants into one group (rather than, e.g., as a continuous measure – this is particularly relevant given that face perception processes are known to change across the tested ages)?

Response: **The Reviewer 1 is right. Indeed, we treated age as a continuous predictor in all statistical models in the first place. However, no effect of age emerged. We tried to clarify this result in the result section of the revised ms pp. 25. Also, we provided data visualization with correlation plot for age and target detection times for both face and scrambled stimuli in Figure D (above reported). No association emerges by looking at changes in target detection times and age.**

- Did the ASD group consist of children of varying mental ages, is there any information at all?

Response: **In addition to the diagnosis, the only information provided by the treatment centres suggest that in our sample children preferentially showed nonverbal than verbal communicative skills. Nevertheless, according to the informed consent the centres did not share additional information with the experimenters. We added this information in the discussion sections p.16.**

- How many valid trials did each group actually complete? It states that the criterion of "less than 2 valid trials for each level of the design" was used to exclude ASD participants, but even two trials would not seem enough for analysis. It would be beneficial to see the number of valid trials per group. Also, 48 trials in total for face/scrambled conditions and valid/ISO/IDO – does this mean 8 trials per level?

Response: **The total number of trials in the two group was similar (ASD = 292; TD = 271) and it had been reported in table 2. Moreover, Figure B. (above reported) shows that no substantial difference emerged between groups on the average number of valid trials per participant across condition and stimulus.**

We adopted as a minimum number of two trials to include a participant in the analysis mainly for reducing participants drop-out. Please consider also that, in children research missing data and "unbalanced repeats" are the norm rather than the exception, and this is the main reason why we chose mixed-effects models that preserve statistical power while dealing with missing data.

Indeed, mixed-effects modelling - that are suitable to estimate effects at the group level and those related to individual differences - did converge successfully suggesting that our sample size (563 observations) was adequate to compute coefficient estimation. Mixed-effects modelling are also suited to analyze higher-order clustered factors (e.g., children nested within groups, trials nested within children) and do not converge when data points are insufficient to compute the model's parameters (Bates et al., 2015).

To further test whether our total number of trials was adequate to estimate reliable coefficients, we compared the model discussed in the original ms to a maximal model with a by-participant random slope for the fixed factor condition which requires a larger number of observations to converge. As shown in Figure E (below reported), the estimated coefficients (and related standard errors) did not show a substantial difference by comparing the two models with the same interaction terms. Importantly, the maximal random effect structure did not impact effect estimates. We revised the method by replacing the estimated parameters computed with the conservative and reliable maximal model, pp. 8-12

Figure E. **The estimates coefficient and relative standard error comparison between the model discussed in the original ms (CONDITION * STIMULUS * GROUP) in red and the model discussed in the revised ms (CONDITION * STIMULUS * GROUP with by-subject random slope for conditions), in black.**

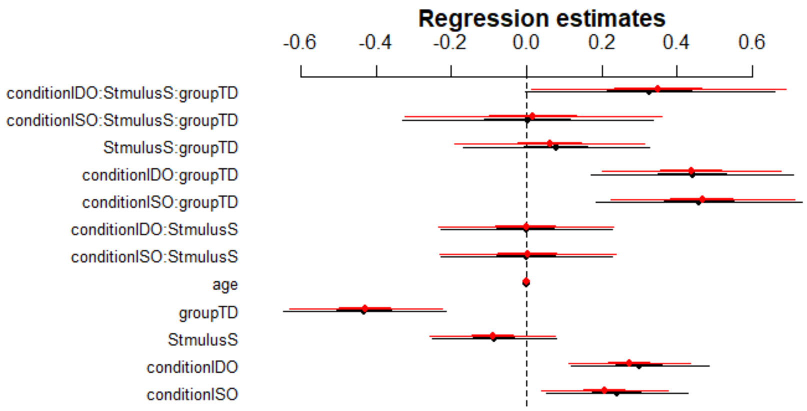

- Why was cue position a factor in the analysis?

Response: **We thank Reviewer 1 for noticing this misleading sentence in the first line of the Design section. 'Cue position' referred to the target location relative to the cue position: that is, our factor condition (i.e., Valid, Same Object and Different Object conditions). We edit and clarify this aspect accordingly in the revised ms p. 8.**

- Why did the 'four quality criteria' not confirm that participants made a saccade to the target by examining, e.g., time taken until entry to the AOI that contained the target (rather than the 40px toward the target)?

Response: **We apologies for our lack of clarity. Indeed, the "40px toward the target AOI" meant exactly "time taken until entry the AOI that contained the target". We apologize for the misleading sentence That has been modified in the Data analysis section accordingly, p.9. Moreover, Figure G. (below reported) precisely shows the AOI area (light-gray squares).**

Figure G. **An example of a trial considered valid. At target onset (yellow circle) the fixation should be found at central fixation (AOI around the green circle). Then, a saccade (the green line) towards the target AOI is typically found.**

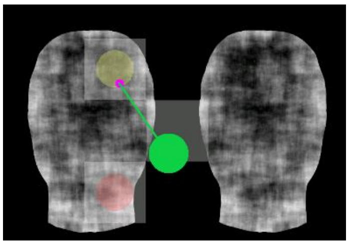

The discussion mainly focused on methodological differences between the present study and previous studies to explain any discrepancies between findings. While this was of course useful and insightful, a deeper discussion of the theoretical implications would be helpful to highlight the significance of the present findings, e.g. how do group differences in object-based attention specifically relate to or explain social attention in ASD, what may be possible developmental mechanisms? Greater emphasis on such a theoretical discussion is required (and could be added as a final sentence in the abstract).

Response: **We thank Reviewer 1's comment we extensively expanded the discussion by addressing the implication that link object-based attention to social attention in ASD pp. 12-13. We did so also thanks to the literature suggested by Reviewer 2. We included a final sentence in the abstract, according to Reviewer 1 comment.**

Minor comments

- A mean effect size of 0.55 was reported (cf. Chita-Tegmark, 2016) but I could not find this value in the original paper

Response: **In the Results section pp. 84, Chita-Tegmark (2016) reported the results of the metanalysis indicating that: "[…]** ***The random effects analysis of overall effect size indicated a mean effect size of 0.55,with 95% confidence limits from 0.38 to 0.73. This is a medium effect size (Cohen, 1988) and indicates that overall, individuals with autism spend less time than typically developing controls attending to social stimuli. […]"***

The author of the metanalysis also refers to the estimated mean effects at the begin of the Discussion section, p. 88: " ***[…] The average Cohen's d effect size across studies was 0.55, a medium effect. […]".***

- It might be beneficial to explain space- vs object-based attention earlier in the manuscript.

Response: **As required, we extensively revised the** ***Section 1.1.*** **making clearer the relevance of the investigation space- vs object-based attention according to the Reviewer's comment, p.5.**

- "uninterpretable eye movements" is an unusual expression – possibly "low-quality eye tracking data" would work if this is what the authors meant.

Response: **We modify the text accordingly p. 5, thank you.**

- It would help the reader if the text referred to the figures when they are described, e.g. when mentioning the sequence of displays in the methods, refer to Fig2.

Response: **As required, we now directly refer to Figure 2 in the methods section.**

- "(POG) was calibrated by presenting an attractive, looming stimulus in 5 positions of the screen […] to validate the accuracy of the calibration" – calibration cannot validate the accuracy; this could be fixed by deleting the part from 'to validate'
Response: **We fixed the inaccurate sentence as suggested by Reviewer 1.**

- Figures and tables should use labels that can be understood without the main text. For Fig3: it would be easier to interpret the data if the y-axis for the TD/ASD groups were identical. For Fig4: Please include information on y-axis units (the ticks should also be equally spaced apart), the meaning of error bars, and the nature of the displayed data points (means?). It

would help to reduce the 4 plots into 2 by overlaying data if possible. Since x-axis represents categorical data, the data points should not be connected by lines.

Response: **We modified figure 3 p. 10, by overlapping the TD and ASD group using the same y-axis and showing averaged target detection times in milliseconds split for levels of the fixed factor Stimulus, i.e., F = face stimuli S = scrambled face stimuli. We also modified figure 4 p. 12, as suggested by Reviewer 1, by specifying the meaning of error bars and data points.**

- Why is the central attention getter displayed simultaneously with the cue?

Response: **This methodological choice was made to constraint participant attention towards the central point and to prevent an overt movement towards the cue, in the first place. This allowed a fair comparison because at the cue onset attention was drawn to the central point in all trials. Importantly, it made more likely to kept constant the attentional spatial distance, not only the actual distance between cue and target across trials, see also Figure G (above). We revised the Procedure accordingly p.7.**

- The section “The present study” was not always easy to understand, mostly because statements were quite general (e.g., “deployment of attention and the structure of selected information”, “focal attentional shifting acts upon one discrete object at a time”, “similar attentional cost to shift attention within the cued face and importantly, between the cue and the un-cued face”, “they enlarged the focus of attention in order to monitor all faces”, etc.). Maybe such statements could be more specific.

Response: **We removed the selected sentences and we extensively revised the introduction and the discussion sections, according to the responses above reported. We believe that the ms is now more focused on the relevance of object-based attention in shedding light on the quality of object representations during attention deployment (Chen, 2012). We think that the editing of a native English language proof-reader improved the clarity of the ms.**

- The paper would need to be proof-read due to typos/grammatical errors.

Response: **We provide a proof-red ms changes are marked with the "Track Changes" function in Microsoft Word.**

Reviewer #2:

The authors present a novel application of Egly's paradigm, first used by Valenza et al. (2014), to compare differences in space- and object-based attention with face versus non-face stimuli, between ASD and TD groups. In a simple and clear task, the authors find a space-based facilitation effect for both groups across both stimulus conditions, and an object-based facilitation effect with face stimuli. They find a difference in performance between the two groups in the IDO condition for phase-scrambled faces: the ASD group, but not TD, demonstrating no object-based cost in attentional shift. The authors suggest that this pattern of results is evidence of autistic groups employing a similar attentional strategy for both face and non-face stimuli, offering a potential route to better understanding of atypical attention to social stimuli often observed in the autistic population. These are an interesting set of initial results, and the finding is a useful addition to the literature. The authors are advised to consult a copy-editor/proof-reader to correct typos and minor grammatical errors. My comments are as follows:

Response: **We answered each comment and, as requested, we provided a proofread ms.**

Introduction:

1. When discussing potential face-processing similarities between ASD and non-ASD populations, the authors only mention studies pertaining to social stimuli, rather than faces. They could consider the following papers pertaining to faces/face-like stimuli –

    1. Shah, P., Gaule, A., Bird, G., & Cook, R. (2013). Robust orienting to protofacial stimuli in autism. *Current Biology*, *23*(24), R1087-R1088
    2. Akechi, H., Stein, T., Kikuchi, Y., Tojo, Y., Osanai, H., & Hasegawa, T. (2015). Preferential awareness of protofacial stimuli in autism. *Cognition*, *143,* 129-134.
    3. McPartland, J. C., Webb, S. J., Keehn, B., & Dawson, G. (2011). Patterns of visual attention to faces and objects in autism spectrum disorder. *Journal of autism and developmental disorders,* *41*(2), 148-157. [Might be a helpful reference as it compares attention to both faces and objects]

Response: **We thank Reviewer 2 for suggesting references of relevant studies, we extensively revised the ms by also referring to those studies in the Introduction of the revised ms p.2-3.**
The associated revisions are marked in yellow.

2. Second page, last paragraph: The authors would need to make the study motivation clearer, "hack" does not make sense. They should also consider "atypical" instead of "dysfunctional" (inclusive language).

Response: **We thank Reviewer 2 for the comments. We removed the sentence and widely revised the introduction pp-4. We also replaced all not-inclusive terms.**

3. Section 1.1: This section might benefit from some re-organisation to make for clearer reading. At the moment one has to go back and forth to be able to grasp the concept. Potentially: 1) briefly introducing the two concepts of space-based and object-based attention, 2) talking about how they are not necessarily discrete mechanisms, 3) introducing

the relevant previous literature, 4) then talking about how the present study incorporates this concept. Perhaps the attention literature could be in a preceding paragraph and the present study section need only be a discussion of the present work.

Response: **We thank Reviewer 2 for the comment. We extensively re-arranged the introduction section 1.1 in particular in pp. 3-4, according to the clever suggestions.**

More specifically, we re-framed the introduction highlighting that heterogeneity of the results and interpretations offered by the literature and the need to better understand: 1) which conditions trigger different attentional strategies in children with a diagnosis of ASD compared with children without a diagnosis of ASD, and 2) if such differences only emerge when attention is focused on the face. We explained why Egly's paradigm might be a relevant tool to understand social attention in ASD, pp. 1-5 (revision are marked in yellow). Indeed this paradigm is suitable for evaluating costs and benefits in dislocating attention toward different attentional focuses. When the attentional focus of visual orienting is a particular region in the visual space (i.e., space-based attention), whatever falls within the focused spatial region, be it an object, a group of objects, or nothing at all, is processed more efficiently than stimuli in non-selected regions (e.g., Cave & Bichot, 1999; Posner, 1980). Conversely, when the attentional focus of visual orienting is an object (i.e. object-based attention), the target of attention is not an arbitrary region, but it is exactly those region that corresponds to candidate objects (Driver & Baylis, 1998; Duncan, 1984). For object-based attention to be deployed, a robust object representation must be established (Chen , 2012). Thus, variables that affect the quality of object representations also influence the degree to which object-based attention is employed. Unlike space-based attention, only object-based attention is a gateway to investigate the complex interplay underling the impact of the selected information (object representation) on the deployment of attentional strategies (Driver, Davis, Russell, Turatto, & Freeman, 2001). So this paradigm evaluates costs and benefits in dislocating attention toward different attentional focuses and we believe that it suits for individuate which conditions and stimuli trigger different attentional strategies in children with a diagnosis of ASD compared to children without a diagnosis of ASD.

Minor comments:

1. Second page, 7th line from top: attentional difficulties are not necessarily "comorbid" with ASD in the way that another disorder would be.

Response: **We removed the sentence and did not refer to 'comorbidity' in order to not misled the reader.**

2. Introduction, second page, 12th line from bottom: ASD and TD abilities not necessarily "identical" as underlying social difficulties may be masked. Preferable to re-phrase this as "showed no differences" or some variation of this.

Response: **We modified the sentence as suggested in p. 14 : "[…] By contrast, no object-based cost emerged in the ASD group, suggesting that children with a diagnosis of ASD use similar attentional strategies to shift attention between faces and not-face stimuli. That is, in the ASD group, the space-based components of visual attention mirror those observed in TD children, whereas the object-based components of attention do not. […]"**

3. When talking about previous literature, the authors could consider using "studies" or "previous literature" rather than "researches".
Response: **We replaced the term "researches" with "study" by following the suggestion of Reviewer 2.**

Methods
Minor Comments:
1. Was recalibration done after each pause, and in the case of participants for whom the task had to be stopped and then restarted?
Response: **Calibration was made at the beginning of the experimental session. Recalibration only occurred whether participant asked for a pause, otherwise the whole experiment relied on the first calibration. We include this information in the procedure of the revised ms, p.7**

2. The authors could briefly explain why they chose to employ phase-scrambled faces as opposed to scrambled faces (as in Valenza et al., 2014), since they have retained other aspects of that study's design.
Response: **We thank Reviewer 2 for catching this important point. Valenza et al. (2014) investigated the impact of face on space-based and object-based attention in 8-month-olds. Likely, the authors choose scrambled faces stimuli to trigger to a similar degree infants' attention toward face and non-face stimuli. That is, scrambled face stimuli present all the discrete elements composing a face by maintaining a similar perceived attractiveness in face and not face stimuli. Moving a step forward, phase-scrambled stimuli used in the present study increased the perceived difference between face and non-face stimuli, by also controlling for perceptual features between face and their phase-scrambled version.**
Indeed, scrambled faces used by Valenza et al. (2014) would have not been suitable to investigate the question under discussion, compared with phase-scrambled stimuli. That is, understanding if face and non-face stimuli trigger similar attentional strategies in term of object-based component of attention deployment in children with, and without, a diagnosis of ASD. On note, children are more likely to be attracted by non-social stimuli compared with infants hence the methodological choice has been made according to the age of interest in the present investigation.

3. Section 2.2 first line: could the authors add the missing word "stimuli", i.e., "Face (F) [stimuli] and their phase-scrambled versions (S)…",
Response: **We modified the section 2.2. first line as suggested by Reviewer 2.**

4. In Table 2, could the authors please include an explanation for “SS” (for e.g., in the row conditionISO:SS)
Response: **We clarify the meaning explanation for “SS” (i.e., scrambled stimulus) in the caption of Table 2.**

5. In Figure 4, could the authors please include the unit of measurement for target detection time (presumably ms as in previous figure). Also, in Figure 4, the distances between the markers on the y-axis do not appear equal, although their numerical values suggest they would be.

Response: **Thanks to comments of the Reviewer 1 and 2, we modified figure 3 by overlapping the TD and ASD group using the same y-axis and showing averaged target detection times in milliseconds split for levels of the fixed factor Stimulus, i.e., F = face stimuli S = phase-scrambled face stimuli. We also modified figure 4 as suggested by Reviewer 1, by specifying the meaning of error bars and data points.**

Discussion
Minor comments:
In general, the arguments made in this section put forth a convincing line of reasoning. The authors are particularly advised to consider copy-editing of this section.

1. Second page, last paragraph, 7th line from bottom: At present it is unclear that this line is referencing the Unruh et al. study. The authors could add for clearer reading, the following addition, “[In that study] adolescents with ASD…”
Response: **We modified the sentence as suggested by the Reviewer 2, p. 14.**

2. Third page, 5th line from top unclear: The authors could add for clarity, “[when] presented with high-interest items”.
Response: **We modified the sentence as suggested the by Reviewer 2, p. 14.**

3. Third page, 9th line from top: The use of “collide with” is unclear. Do the authors mean contradict?
Response: **We thank the Reviewer 2, we replaced “contradict” instead of “collide with”.**

4. Third page, last paragraph: It is unclear here which sample population the authors are referring to. The authors would need to clarify this in the first sentence (“it is required to go through…”) and the next (“eye-avoidance/aversion effect…”).

Response: **We thank the Rewiever 2, we disambiguated this point by directly referring to the group of children with a diagnosis of ASD. Moreover we rephrased the sentence also with the help of a professional proof-reader (marked with the "Track Changes" function in Microsoft Word).**
