# Supplementary material for: Attentional shift within and between faces: Evidence from children with and without a diagnosis of autism spectrum disorder
Source: PLoS One. 2021 May 14;16(5):e0251475. doi: 10.1371/journal.pone.0251475 (PMC8121363; doi:10.1371/journal.pone.0251475)
Supplement: Attachment — Submitted filename: Review_PONE-D-20-35397.docx [file pone.0251475.s004.docx]

Review for PONE-D-20-35397

The authors present a novel application of Egly’s paradigm, first used by Valenza et al. (2014), to compare differences in space- and object-based attention with face versus non-face stimuli, between ASD and TD groups. In a simple and clear task, the authors find a space-based facilitation effect for both groups across both stimulus conditions, and an object-based facilitation effect with face stimuli. They find a difference in performance between the two groups in the IDO condition for phase-scrambled faces: the ASD group, but not TD, demonstrating no object-based cost in attentional shift. The authors suggest that this pattern of results is evidence of autistic groups employing a similar attentional strategy for both face and non-face stimuli, offering a potential route to better understanding of atypical attention to social stimuli often observed in the autistic population. These are an interesting set of initial results, and the finding is a useful addition to the literature. The authors are advised to consult a copy-editor/proof-reader to correct typos and minor grammatical errors. My comments are as follows:

Introduction:

1. When discussing potential face-processing similarities between ASD and non-ASD populations, the authors only mention studies pertaining to social stimuli, rather than faces. They could consider the following papers pertaining to faces/face-like stimuli –

1. Shah, P., Gaule, A., Bird, G., & Cook, R. (2013). Robust orienting to protofacial stimuli in autism. *Current Biology*, *23*(24), R1087-R1088
2. Akechi, H., Stein, T., Kikuchi, Y., Tojo, Y., Osanai, H., & Hasegawa, T. (2015). Preferential awareness of protofacial stimuli in autism. *Cognition*, *143*, 129-134.
3. McPartland, J. C., Webb, S. J., Keehn, B., & Dawson, G. (2011). Patterns of visual attention to faces and objects in autism spectrum disorder. *Journal of autism and developmental disorders*, *41*(2), 148-157. [Might be a helpful reference as it compares attention to both faces and objects]

2. Second page, last paragraph: The authors would need to make the study motivation clearer, “hack” does not make sense. They should also consider “atypical” instead of “dysfunctional” (inclusive language).

3. Section 1.1: This section might benefit from some re-organisation to make for clearer reading. At the moment one has to go back and forth to be able to grasp the concept. Potentially: 1) briefly introducing the two concepts of space-based and object-based attention, 2) talking about how they are not necessarily discrete mechanisms, 3) introducing the relevant previous literature, 4) then talking about how the present study incorporates this concept. Perhaps the attention literature could be in a preceding paragraph and the present study section need only be a discussion of the present work.

Minor comments:

1. Second page, 7^th^ line from top: attentional difficulties are not necessarily “comorbid” with ASD in the way that another disorder would be.

2. Introduction, second page, 12^th^ line from bottom: ASD and TD abilities not necessarily “identical” as underlying social difficulties may be masked. Preferable to re-phrase this as “showed no differences” or some variation of this.

3. When talking about previous literature, the authors could consider using “studies” or “previous literature” rather than “researches”.

Methods

Minor Comments:

1. Was recalibration done after each pause, and in the case of participants for whom the task had to be stopped and then restarted?

2. The authors could briefly explain why they chose to employ phase-scrambled faces as opposed to scrambled faces (as in Valenza et al., 2014), since they have retained other aspects of that study’s design.

3. Section 2.2 first line: could the authors add the missing word “stimuli”, i.e., “Face (F) [stimuli] and their phase-scrambled versions (S)…”,

4. In Table 2, could the authors please include an explanation for “SS” (for e.g., in the row conditionISO:SS)

5. In Figure 4, could the authors please include the unit of measurement for target detection time (presumably ms as in previous figure). Also, in Figure 4, the distances between the markers on the y-axis do not appear equal, although their numerical values suggest they would be.

Discussion

Minor comments:

In general, the arguments made in this section put forth a convincing line of reasoning. The authors are particularly advised to consider copy-editing of this section.

1. Second page, last paragraph, 7^th^ line from bottom: At present it is unclear that this line is referencing the Unruh et al. study. The authors could add for clearer reading, the following addition, “[In that study] adolescents with ASD…”

2. Third page, 5^th^ line from top unclear: The authors could add for clarity, “[when] presented with high-interest items”.

3. Third page, 9^th^ line from top: The use of “collide with” is unclear. Do the authors mean contradict?

4. Third page, last paragraph: It is unclear here which sample population the authors are referring to. The authors would need to clarify this in the first sentence (“it is required to go through…”) and the next (“eye-avoidance/aversion effect…”).
