# Supplementary material for: Attentional shift within and between faces: Evidence from children with and without a diagnosis of autism spectrum disorder
Source: PLoS One. 2021 May 14;16(5):e0251475. doi: 10.1371/journal.pone.0251475 (PMC8121363; doi:10.1371/journal.pone.0251475)
Supplement: Attachment — Submitted filename: Response_to_Reviewers.docx [file pone.0251475.s006.docx]

6. Review Comments to the Author

Reviewer #1: The revised manuscript is greatly improved, and the authors have addressed my previous comments in a satisfactory manner. The updated structure of the introduction lays out a coherent rationale of the study aims and Egly’s paradigm specifically. The methods are also much clearer. I have a couple of final, very minor comments/suggestions.

- “comorbid attentional atypicalities”: remove ‘comorbid’ since this does not relate to a clinical condition
- Table 2/Fig 3: typo in “Stimulus”
- “challenging the notion that a primitive sensitivity to protoface stimuli is essential for typical social development”: I think the authors meant ‘sufficient’ rather than ‘essential’ (the terms have different meanings). It also seems that the following sentence aims to express the same idea.

**Response: We thanks Reviewer 1 for appreciating the current version of the ms. We fixed the typos accordingly. Changes are marked with the Track changes function of MS word in the ms.**

- “whether such differences only emerge when attention is focused on the face”: it may be useful to clarify ‘attention is focused on the face’ since this could be interpreted in different ways depending on a reader’s academic background and their interpretation of 'attentional focus'

**Response: We clarified the sentence as follow: *“[…] whether such differences only emerge when infants pay attention to face. […]*”**

- Thank you for explaining the age-matching procedure. It sounds like individuals were matched on both age and sex (as also stated in the Discussion), in which case it should be added in the methods for clarification, i.e., in the sentence “Participants diagnosed with ASD and TD that meet the inclusion criteria were matched on chronological age.”

**Response: We added this information in the methods as follow: “*Participants diagnosed with ASD and TD that meet the inclusion criteria were matched on chronological age and sex*.”**

- Thank you for clarifying the quality criteria. It seems the authors adopted my phrasing (“time taken until entry the AOI that contained the target”), but in the context of the ms I would suggest something along the lines of: ‘d) participants’ gaze entered the AOI that contained the target’ - I assume this is what was meant.

**Response: Thank you, we revised the sentence accordingly.**

- The updated figures are much easier to interpret and highlight the findings, thank you. Are Fig 3 and 4 displaying the same dataset but as individual points (Fig3) vs summary stats + error bars (Fig4)? If yes, I wonder if the graphs can be combined, although not a necessity. Individual points and summary stats are both useful, but if figures show the same data then it could be confusing as to why separate figures were generated.

**Response: We appreciated the suggestion of the Reviewer 1. We combined Fig3 and Fig4 in a unique figure (i.e. Fig.3). We combined the figure’ captions accordingly by indicating two separate sections i.e., a) and b).**

- “reduce confusion in the estimation…”: not clear what is meant by this part

**Response: We clarify the meaning of the sentence as follow: “*This evidence stresses the importance of carefully choosing non-social stimuli to clearly disambiguate between the impact of social and non-social stimuli on attention, in individuals with ASD*.”**

- “collide with”: contrast?
- “we found a quite remarkable heterogeneity”: for the ms, it might be better written as “we observed greater variability in the data from the ASD group compared to the TD group”, or similar.
- “huge variability”: replace huge with ‘high’
- “Continuos”: typo, ‘continuous’

**Response: We thank the Reviewer 1 to carefully going through stylistic issues in the previous version of the ms. We corrected the submitted ms revision accordingly.**

Reviewer #2: The manuscript is greatly improved. The introduction now reads clearly, and better sets up the premise of the experiment; particularly, the discussion on space-based vs object-based attention and why Egly’s paradigm is a suitable choice for this study. The methods and accompanying figures are more intelligible. The discussion better highlights the implications of the present set of results.

My comments are as follows:

1. Page 2, ref [20] and [21]: “On the one hand, to explain why people with ASD spend less time with attention on faces, some authors have proposed that this might derive from an innate atypicality of the face detection mechanism, that is the subcortical mechanism that tunes infant attention to face-like stimuli from birth [20]. However, evidence indicates that individuals with ASD exhibit entirely typical orienting responses to face-like stimuli, challenging the notion that a primitive sensitivity to protoface stimuli is essential for typical social development [21].”
Please note that reference [20] (Shah, Gaule, Bird, & Cook 2013) proposes that the robust orienting effect towards proto-face stimuli in the ASD population which they find speaks against developmental accounts which suggest that reduced looking behaviour in ASD is a consequence of atypical face detection mechanisms. Accordingly, please cite appropriate references to support the first sentence and please include reference [20] along with reference [21].

**Response: We thank the Reviewer 2 to pointing out this reference typo. We modified the reference list by substituting the reference [20] with the study by Di Giorgio, et al.. (2016). Difference in visual social predispositions between newborns at low-and high-risk for autism. Scientific reports, 6(1), 1-9.**

**We update the references list accordingly, hence in the revised ms the study by Shah, Gaule, Bird, & Cook 2013 refers to [21] as in the sentence: “*However, evidence indicates that individuals with ASD exhibit entirely typical orienting responses to face-like stimuli, challenging the notion that a primitive sensitivity to protoface stimuli is essential for typical social development [21]”.***

2. Page 2: “Accordingly, several studies reported that individuals with a diagnosis of ASD show comorbid attentional atypicalities involving the alerting network”.
Attentional difficulties are not necessarily “comorbid” with ASD in the way that another disorder would be. Please remove “comorbid”, the sentence will then read: … “individuals with a diagnosis of ASD show attentional atypicalities involving the alerting network”.

**Response: We removed the term “comorbid” accordingly, thank you. Changes are marked in the text with the Track changes function of MS word.**

3. Page 3: “To answer these questions, we employed a paradigm suitable for evaluating costs and benefits in dislocating attention toward different attentional focuses.”
The authors make the argument for space-based and object-based attention lying on a continuum. In which case, might it be more suitable to replace “dislocating” with “deploying” or another similar word.

**Response: We corrected the revised ms as suggested, thank you.**

4. Page 3: “That is, space-based and object-based attention lie on a continuum rather than be different discrete components of the orienting of attention.”
Instead of “be”, “being” might read better.

**Response: We corrected the revised ms as suggested, thank you.**

5. Page 3, last para: “However, unlike space based attention, only object-based attention is a gateway to investigating the complex interplay [of which processes?] underling the impact of the selected information (object representation) on the deployment of attentional strategies”.
“Complex interplay” implies that two or more processes are involved, which is not made clear currently. Is “underling” perhaps “underlying”?

**Response: We thank the Reviewer 2 for the careful reading of the manuscript, we modified the sentence as follow: *“However, unlike space-based attention, only object-based attention is a gateway to investigating the impact of the selected information (object representation) on the deployment of attentional strategies [41].”***

6. Page 4, present study: “In the current study we compared [which groups?]”.
It would be good to state which sample groups were included at the beginning.

**Response: We clarify the sentence as follow: *“In the current study, we compared the ability to shift attention both toward spatial locations (space-based attention) and objects (object-based attention) in children with, and without, a diagnosis of ASD.”***

7. Page 9, data analysis: “time taken until entry [to] the AOI that contained the target”.
Please include the missing word “to”.

**Response: We corrected the revised ms as suggested by the Reviewer 1, as follow: *“(d) participants’ gaze entered the AOI that contained the target.*”**

8. Page 11: “However, in contrast to the TD group, in the ASD group no cost emerged in attention shift between- vs within-objects” [for both face and phase-scrambled stimuli].
Perhaps this could this be added to the sentence to summarise results better?

**Response: We corrected the revised ms as suggested, thank you.**

9. Page 13, discussion: “Crucially, [in which trials?], stimulus processing is not influenced by the type of the stimulus, but only by the correspondence between the position of the cue and those of the target.
Please could the authors specify in which trials this correspondence is expected to influence performance, i.e., is this the case for only valid trials or all trials facilitated by space-based attention? Presumably the latter, but this would need to be made clear please.

**Response: We thank the Reviewer 2 for the suggestion. We tried to better explain this sentence as follow: *“Crucially in all conditions stimulus processing is not influenced by the type of the stimulus, but only by the position of the cue and that of the target***”.
