# Supplementary material for: Attentional shift within and between faces: Evidence from children with and without a diagnosis of autism spectrum disorder
Source: PLoS One. 2021 May 14;16(5):e0251475. doi: 10.1371/journal.pone.0251475 (PMC8121363; doi:10.1371/journal.pone.0251475)
Supplement: Attachment — Submitted filename: Response_to_Reviewers.docx [file pone.0251475.s007.docx]

6. Review Comments to the Author

**Reviewer #1:** The authors have responded to my comments in a satisfactory manner. I have one last, very minor comment and otherwise only noted down some typos (it may be useful to do a final proof-read).

Comment: p.3, ‘emerge when infants pay attention to face’ - should be corrected to 'to the face'. Secondly, I am not sure why the authors revised this to refer to infants specifically. I would replace with ‘individuals’ or 'children' if this is what the authors meant.

Typos:
- p.2: “confirm these pattern of data” change to “confirm these patterns of data”
- p.4: “rest of the object benefit” change to “rest of the object benefits”
- p.6: “accordance with the principles laid down in the Declaration of Helsinki” change to “accordance with the Declaration of Helsinki”
- p.9: “detection time for each trials” change to “detection time for each trial”
- p.13: “but only by the the position”; delete 1x ‘the’.

**Author**: We thank Reviewer 1, we carefully edited the ms as suggested. Please find all changes marked by the track-changes function of MS in the revised ms.
